# A randomized trial of transdermal and oral estrogen therapy in adolescent girls with hypogonadism

**DOI:** 10.1186/1687-9856-2014-12

**Published:** 2014-06-20

**Authors:** Sejal Shah, Nikta Forghani, Eileen Durham, E Kirk Neely

**Affiliations:** 1 Division of Pediatric Endocrinology and Diabetes, Stanford University, Stanford CA (S.S., E. D., E.N.), 300 Pasteur Drive, G-313, 94305 Stanford, CA, USA; 2 Pediatric Endocrinology and Diabetes, Children’s Hospital of Orange County, Orange CA (N.F.), 1201 W La Veta, 92868 Orange, CA, USA

**Keywords:** Puberty, Female, Feminization, Hormone replacement therapy, Clinical trial, Estrogen

## Abstract

**Background:**

Adolescent females with ovarian failure require estrogen therapy for induction of puberty and other important physiologic effects. Currently, health care providers have varying practices without evidence-based standards, thus investigating potential differences between oral and transdermal preparations is essential. The purpose of this study was to compare the differential effects of treatment with oral conjugated equine estrogen (OCEE), oral 17β estradiol (OBE), or transdermal 17β estradiol (TBE) on biochemical profiles and feminization in girls with ovarian failure.

**Study design:**

20 prepubertal adolescent females with ovarian failure, ages 12–18 years, were randomized to OCEE (n = 8), OBE (n = 7), or TBE (n = 5) for 24 months. Estrogen replacement was initiated at a low dose (0.15 mg OCEE, 0.25 mg OBE, or 0.0125 mg TBE) and doubled every 6 months to a maximum dose of 0.625 mg/d OCEE, 1 mg/d OBE, or 0.05 mg/d TBE. At 18 months, micronized progesterone was added to induce menstrual cycles. Biochemical markers including sex hormones, inflammatory markers, liver enzymes, coagulation factors, and lipids were obtained at baseline and 6 month intervals. Differences in levels of treatment parameters between the groups were evaluated with one-way analysis of variance (ANOVA). The effect of progesterone on biochemical markers was evaluated with the paired *t*-test.

**Results:**

Mean (±SE) estradiol levels at maximum estrogen dose (18 months) were higher in the TBE group (53 ± 19 pg/mL) compared to OCEE (14 ± 5 pg/mL) and OBE (12 ± 5 pg/mL) (p ≤ 0.01). The TBE and OBE groups had more effective feminization (100% Tanner 3 breast stage at 18 months). There were no statistical differences in other biochemical markers between treatment groups at 18 months or after the introduction of progesterone.

**Conclusions:**

Treatment with transdermal 17β estradiol resulted in higher estradiol levels and more effective feminization compared to oral conjugated equine estrogen but did not result in an otherwise different biochemical profile in this limited number of heterogeneous patients. OBE and TBE provide safe and effective alternatives to OCEE to induce puberty in girls, but larger prospective randomized trials are required.

**Trial registration:**

Clinical Trials Identifier: NCT01023178.

## Background

The use of estrogen in girls with ovarian failure is necessary to induce puberty, cause feminization and deliver other important physiologic effects. It is apparent that pediatric endocrinologists have widely varying practices regarding the type and dose of estrogen used for the feminization of hypogonadal girls [1,2]. Ultimately, since adolescents with ovarian failure will need estrogen therapy for a prolonged period of time, investigating potential differences between oral and transdermal preparations is important and necessary. Several studies have compared estrogen preparations in adult females, but the adolescent population is relatively understudied. A recent study in girls with TS demonstrated more physiologic estrogen concentrations with the use of the transdermal estrogen preparation [1].

Classically, estrogen has been thought to have cardio-protective effects. In hypogonadal young adult women increasing doses of oral estrogen are associated with decreasing intima media thickness, increasing high-density lipoprotein (HDL) levels, and decreasing plasma glucose levels [3]. These effects need to be weighed against the potentially prothrombotic effects of estrogen highlighted by the Women’s Health Initiative (WHI) [4]. Some have theorized that the higher frequency of adverse events in the WHI reflects the type of estrogen used in these studies, oral conjugated equine estrogen (OCEE). Furthermore, other adverse effects associated with use of OCEE in adults, such as elevation of c-reactive protein, liver enzymes, increases in pro-coagulation factors, hypertension, decreases in HDL cholesterol, and increases in triglycerides, have not been seen with transdermal 17β estradiol (TBE) [5-11]. Use of oral 17β estradiol (OBE) in adult females has had variable effects [12], suggesting that OCEE itself may have inflammatory or metabolic effects independent of the route of estrogen delivery, as it is composed of many different forms of estrogen (primarily estrone).

The need for estrogen replacement in adolescence is not limited to girls with TS. Lantiga et al. showed that 18% of female childhood cancer survivors will develop ovarian failure by age 33, necessitating long-term estrogen use [13]. Other studies have shown that the incidence of impaired ovarian function or frank failure is up to 85% of cancer survivors after bone marrow transplant [14]. Furthermore, adolescent cancer survivors with ovarian failure often have baseline liver damage due to their initial treatment regimen, and using a less hepatotoxic form of estrogen could have important clinical significance.

In addition to estrogen replacement therapy, progesterone therapy is required to induce menstrual cycles. There is a lack of studies exploring the metabolic effects of adding progesterone to estrogen therapy in girls. The Heart Estrogen/Progesterone Replacement study (HERS) and WHI studies highlighted the deleterious effects of progesterone in adults, as combined therapy resulted in higher rates of stroke and other cardiovascular disease [15-17].

This randomized, open-label feasibility study was designed to investigate the differential effects of treatment with three common forms of estrogen on the biochemical profiles in hypogonadal girls, as well as to evaluate the discrete effects of progesterone therapy. We hypothesized that transdermal estrogen would have less harmful biochemical effects than oral forms of estrogen, and that there would be differences between the biochemical profiles of the two oral forms of estrogen, OCEE and OBE. Additionally, we hypothesized there would be no differences in feminization among all forms of estrogen replacement.

## Methods

### Subjects

All adolescent females between the ages of 12–18 years with primary or secondary ovarian failure in whom estrogen therapy was clinically indicated were invited to participate in the randomized open-label study. During a 4-year period (2007–2011), 25 study participants were assessed for eligibility, recruited from current patients and new referrals in the Pediatric Endocrinology Clinic at Lucile Packard Children’s Hospital at Stanford. All eligible patients seen in the clinic were approached to participate. Twenty patients were enrolled in the study. We excluded girls who had Tanner 2 breast stage or greater, spontaneous menses, or previous use of estrogen. Written informed consent was obtained from parents of all participants before enrollment, and a written assent was obtained from all study participants. The protocol and consents were approved by the Stanford University Administrative Panel on Human Subjects in Medical Research.

### Protocol

Participants were randomized to one of 3 forms of estrogen: OCEE (Premarin, Wyeth Pharmaceuticals, Madison, New Jersey), OBE (Estrace, Warner Chilcott, Dublin Ireland) or TBE (Vivelle-Dot, Novartis, East Hanover, New Jersey). Block randomization was used, with sequentially numbered envelopes that contained treatment group assignments. Participants and care providers were not blinded to the treatment groups. Estrogen replacement was initiated at a low dose (0.15 mg/d OCEE, 0.25 mg/d OBE, or 0.0125 mg/d TBE) and doubled every 6 months to a maximum dose starting at 12 months of 0.625 mg/d OCEE, 1 mg/d OBE, or 0.05 mg/d TBE. These doses were selected based on published equivalencies at the start of the study [18]. At 18 months, micronized progesterone (Prometrium, Abbott, Abbott Park, Illinois) at a dose of 200 milligrams daily was introduced on days 17–26 of each month, and subjects were asked to stop both estrogen and progesterone on days 27–31 of each month to induce a menstrual cycle. Study participants were followed for 6 months after the introduction of progesterone, for a total study time of 24 months.

### Evaluation and laboratory testing

History and physical exams, including assessment of feminization by Tanner staging and breast diameter were obtained at baseline and every 6 months for 24 months. The biochemical profile was obtained as a morning fasting venous sample at baseline and 6 month intervals that coincided with the subject’s clinic visits. There was not a specific attempt to avoid sampling during illnesses or during menses. Each sample included biochemical markers of inflammation (high-sensitivity c-reactive protein [hs-CRP] and fibrinogen), sex hormones (estradiol, estrone, estrone sulfate, luteinizing hormone [LH], follicle-stimulating hormone [FSH]), liver enzymes and binding proteins (alanine aminotransferase [ALT], aspartate aminotransferase [AST], sex hormone binding globulin), lipid metabolism (cholesterol, HDL, low-density lipoprotein [LDL], triglycerides), coagulation (antithrombin activity), insulin resistance (insulin, fasting glucose), growth (insulin-like growth factor -1 [IGF-1] and insulin-like growth factor binding protein-3 [IGFBP-3]), and blood pressure (plasma renin activity). All assays were performed at Esoterix (Calabasas Hills, California). The hs-CRP assay was performed by nephelometry. The estradiol assay was performed by liquid chromatography/tandem mass spectrometry (LC-MS/MS) with a lower level of detection of 1 pg/mL.

### Statistics

Differences in levels of treatment parameters between the three groups were evaluated with one-way analysis of variance (ANOVA). Results are displayed as mean ± SE unless otherwise noted. The comparison within groups between baseline and 18 months was evaluated with a *t*-test, and the effect of progesterone on biochemical markers was evaluated with paired *t*-test. All statistical analysis was performed using SAS Enterprise Guide version 4.3 (SAS Institute, Cary, North Carolina). Subjects with hs-CRP level greater than 10 mg/L were excluded from analysis of the inflammatory marker at that study time point as hs-CRP levels above this cutoff generally suggest an acute systemic inflammatory condition and thus cannot be used to predict cardiovascular risk [19]. One subject with diabetes mellitus was excluded from analysis of glucose and insulin levels. One subject was excluded from all height analysis due to inability to obtain accurate standing heights. Subject data were excluded from analysis of all data obtained at a specific study visit if non-adherence to therapy was reported.

## Results

**Figure 1:**
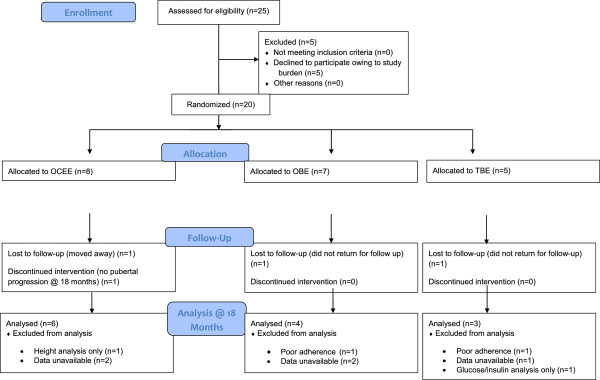
Enrollment, randomization, and follow-up of the study participants.

Twenty adolescent females were enrolled during the study time period (OCEE n = 8; OBE n = 7; TBE n = 5) (Figure 1, Table 1). There were no significant differences in baseline age, height, and weight when comparing the 3 treatment groups. Twelve out of 20 participants had TS (OCEE n = 4; OBE n = 5; TBE n = 3), while the remainder had primary or secondary ovarian failure from pituitary disease or chemotherapy. As can be observed in Table 1, the groups included both hypergonadotropic and hypogonadotropic subjects. At study onset 12 subjects (11 subjects with TS) were treated with recombinant growth hormone (rGH).

**Table 1:** Baseline clinical characteristics of study subjects

	**Group**	**Age (y)**	**Height SDS**	**BMI (kg/m**^**2**^**)**	**Diagnosis**	**Years on GH**	**FSH**	**LH**
	OCEE	15	-1.065	15.3	Kallmann syndrome	-	2	0.47
	OCEE	14.27	-3.835	13.2	BMT	-	194	86
	OCEE	12.98	-1.211	26.2	BMT	-	117	51
	OCEE	15.21	-1.678	40.2	Turner syndrome	-	55	29
	OCEE	13.31	-2.922	19.0	Turner syndrome	3.5	58	13
	OCEE	14.36	-1.577	19.3	Panhypopituitarism	5	0.027	0.009
	OCEE	12.9	-2.997	22.2	Turner syndrome	1.7	65	16
	OCEE	12.56	-1.866	20.1	Turner syndrome	2.3	197	15
*Mean ± SE*		*13.8 ± 0.4*	*-2.14 ± 0.35*	*22 ± 3*		*3.1 ± 0.7*	*86 ± 27.2*	*26 ± 10.3*
	OBE	16.11	0.061	18.5	Kallmann syndrome	-	0.656	0.041
	OBE	13.62	-3.141	18.2	AI	-	144	14
	OBE	14.15	-1.532	20.0	Turner syndrome	9.4	46	12
	OBE	16.05	-4.203	17.0	Turner syndrome	0.8	117	26
	OBE	14.3	-2.723	20.5	Turner syndrome	3.7	40	8.5
	OBE	15.32	-4.486	19.4	Turner syndrome	1.7	70	16
	OBE	12.02	-1.608	19.1	Turner syndrome	0.7	76	13
*Mean ± SE*		*14.5 ± 0.6*	*-2.52 ± 0.61*	*19 ± 0.4*		*3.3 ± 1.6*	*71 ± 18.3*	*13 ± 3*
	TBE	15.32	0.291	16.6	Kallmann syndrome	-	1.5	0.15
	TBE	13.75	-2.637	13.0	Delayed puberty	-	3	0.571
	TBE	12.5	-3.03	19.1	Turner syndrome	5	118	24
	TBE	15.43	-3.079	18.5	Turner syndrome	0.8	114	17
	TBE	14.16	-3.742	19.5	Turner syndrome	7.3	144	26
*Mean ± SE*		*14.2 ± 0.5*	*-2.44 ± 0.71*	*17.4 ± 1.2*		*4.4 ± 1.9*	*76 ± 30.6*	*14 ± 5.6*

Biochemical and growth data collected at baseline are shown by treatment group in Table 2. Mean estradiol levels were in the prepubertal range (overall mean 3 ± 1 pg/mL). There were no significant differences in baseline biochemical markers between the 3 groups, except for serum glucose concentration, which was not considered clinically significant. Biochemical and growth data after 18 months of estrogen-only therapy are shown in Table 3. The mean estradiol level in the TBE group at 18 months (53 ± 19 pg/mL, *p* ≤ 0.01) was significantly higher compared to subjects treated with both types of oral estrogen. Estrone and estrone sulfate levels in the OBE group trended higher than in the other groups. There were no statistical differences between treatment groups at 18 months in gonadotropins, markers of inflammation, lipid metabolism, growth, insulin resistance, liver enzymes, and renin.

**Table 2:** Treatment parameters at baseline (Mean ± SE)

	**Oral conjugated E2**	**Oral 17β E2**	**Transdermal 17β E2**
Estradiol (pg/mL)	3.1 ± 1.1	1.2 ± 0.3	5.2 ± 3.5
Estrone (pg/mL)	11.3 ± 4.2	5.1 ± 0.8	9.3 ± 2.4
Estrone Sulfate (ng/dL)	18 ± 6.5	10 ± 1.8	11 ± 0.9
FSH (mIU/mL)	86 ± 27.2	71 ± 18.3	76 ± 30.6
LH (mIU/mL)	26 ± 10.3	13 ± 3	14 ± 5.6
SHBG (nmol/L)	63 ± 22	52 ± 11	67 ± 13.4
HS-CRP (mg/L)	1.5 ± 0.9	2.6 ± 2.1	1.5 ± 1.2
Fibrinogen Activity (mg/dL)	305 ± 29.2	280 ± 25.3	291 ± 30.6
Antithrombin Activity (%)	115 ± 4.4	125 ± 5	120 ± 4
Glucose (mg/dL)	74 ± 2.7^*a*^	85 ± 2.8	84 ± 1.7
Insulin (uIU/mL)	7.2 ± 2	5.1 ± 1.3	4.5 ± 1
AST (U/L)	39 ± 8.2	25 ± 2.2	25 ± 0.8
ALT (U/L)	46 ± 18.7	22 ± 3.6	16 ± 2.1
Cholesterol (mg/dL)	187 ± 15.3	168 ± 7.2	167 ± 11.1
Triglycerides (mg/dL)	140 ± 21.8	76 ± 13.9	101 ± 16.3
HDL (mg/dL)	49.3 ± 4	55 ± 6.2	49 ± 5.8
LDL (mg/dL)	113 ± 16.1	94 ± 5.1	97 ± 7.9
Plasma Renin (ng/dL/h)	192 ± 41.4	230 ± 71	229 ± 78.7
IGFBP-3 (mg/L)	3.1 ± 0.5	3.9 ± 0.2	4 ± 0.6
IGF-1 (ng/mL)	226 ± 50.2	361 ± 62	318 ± 84

^*a*^*p* < 0.05, differences in mean values between groups at baseline

**Table 3:** Treatment parameters at maximum estrogen dose (18 Months) (Mean ± SE)

	**Oral conjugated E2 (0.625 mg/d)**	**Oral 17β E2 (1 mg/d)**	**Transdermal 17β E2 (0.05 mg/d)**
Weight (kg)	53.8 ± 10.1	43.6 ± 4	40.6 ± 3.1
Change in Weight from baseline (kg)	5.9 ± 0.7	7.1 ± 1.6	7.2 ± 0.8
Height (cm)	153.2 ± 2.9	147.6 ± 3.9	148.6 ± 3.5
Change in Height from baseline (cm)	6.3 ± 1.3	8.1 ± 1.3	8.2 ± 1.7
Estradiol (pg/mL)	14 ± 5^*a*^	12 ± 5	53 ± 19^*a,b*^
Estrone (pg/mL)	57.7 ± 22.6^*a*^	53.4 ± 28	26 ± 4^*a*^
Estrone Sulfate (ng/dL)	209 ± 68.1^*a*^	364.8 ± 153^*a*^	72 ± 27.9^*a*^
FSH (mIU/mL)	30 ± 9.4	88 ± 25.6	29 ± 26.4
LH (mIU/mL)	29 ± 14.5	44 ± 13.7^*a*^	4.8 ± 3.7
SHBG (nmol/L)	130 ± 38.3	35.5 ± 7.4	59 ± 20.2
HS-CRP (mg/L)	6.27 ± 4	0.5 ± 0.2	0.1 ± 0.03
Fibrinogen Activity (mg/dL)	347 ± 59.8	284 ± 20.2	205 ± 38.9
Antithrombin Activity (%)	109 ± 3.2	121 ± 5.4	102 ± 5
Glucose (mg/dL)	82 ± 5.5	83 ± 3.8	82 ± 0
Insulin (uIU/mL)	19.6 ± 12.8	5.5 ± 1.2	3.9 ± 0.7
AST (U/L)	47 ± 13.6	24 ± 3.3	19 ± 1.2
ALT (U/L)	55 ± 26.6	23 ± 5.3	15 ± 3.5
Cholesterol (mg/dL)	177 ± 18.7	160 ± 0.6	155 ± 19.1
Triglycerides (mg/dL)	195 ± 72.4	126 ± 42.3	62 ± 5.5
HDL (mg/dL)	54 ± 5.1	49 ± 6	54 ± 10.7
LDL (mg/dL)	95 ± 11.2	91 ± 5.8	85 ± 11.3
Plasma Renin (ng/dL/h)	313 ± 83	178 ± 46	243 ± 103.5
IGFBP-3 (mg/L)	2.9 ± 0.4	3.6 ± 0.4	3.2 ± 0.1
IGF-1 (ng/mL)	266 ± 73.1	482 ± 73.6	355 ± 71

^*a*^*p* < 0.05, differences in mean values within groups, baseline vs 18 months.

^*b*^*p* < 0.05, differences in mean values between groups at 18 months.

**Figure 2:**
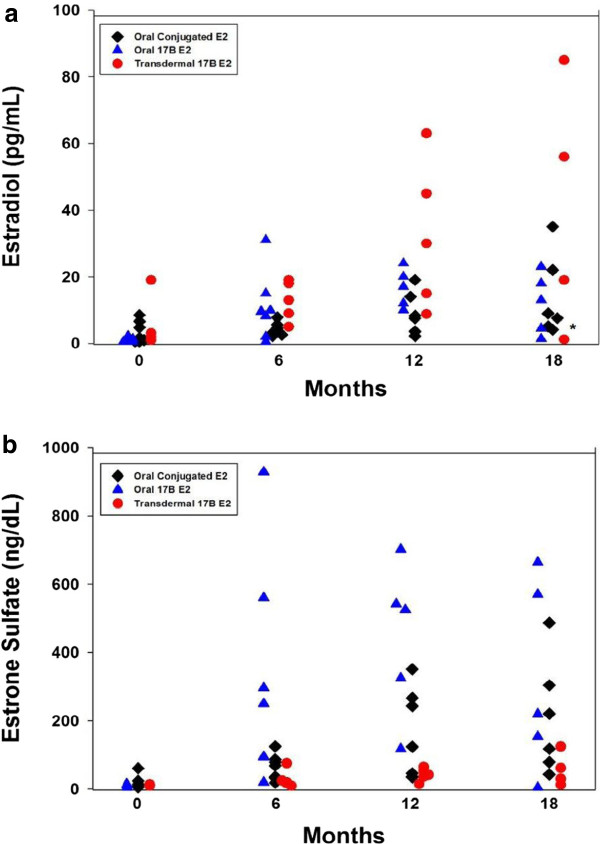
**Estradiol (panel a) and estrone sulfate (panel b) from baseline through 24 months of estrogen-alone therapy in patients treated with oral conjugated estrogen (OCEE), oral 17β estradiol (OBE) or transdermal 17β estradiol (TBE).** (*) TBE, OBE subject with reported non-adherence.

**Figure 3:**
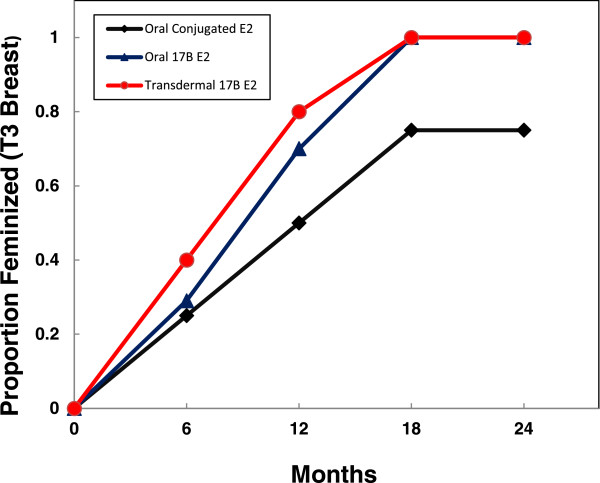
Proportion of subjects in each treatment group to reach Tanner breast stage 3 assessed at each study interval.

Trends in estradiol levels at 6 month intervals are shown in Figure 2a. Higher estradiol levels are noted in the TBE group compared to OBE and OCEE but with large variability within the groups. All groups demonstrated a trend of increasing estradiol levels throughout the estrogen-only portion of the study. A larger percentage of subjects in the TBE group had estradiol levels > 20 pg/mL. Trends towards higher estrone sulfate levels were seen in OBE subjects throughout the estrogen-only treatment period (Figure 2b). Mean estrone sulfate level at 12 months was significantly higher in subjects treated with OBE (378 ± 99 ng/dL, *p* < 0.01) compared to subjects treated with OCEE or TBE (157 ± 49 ng/dL and 42 ± 8 ng/dL, respectively), and a similar trend was apparent at 18 months as well.The proportion of subjects in each group reaching Tanner 3 breast stage at each 6 month interval is shown in Figure 3. All subjects in the TBE and OBE group were feminized by 18 months, whereas only 75% (6/8) of subjects in the OCEE group achieved Tanner 3 breast stage before or 6 months after the scheduled introduction of progesterone.

To evaluate the effect of progesterone therapy, biochemical markers were reassessed after 6 months of estrogen plus progesterone therapy. Participants from the three estrogen groups whose data were available for both month 18 and month 24 were pooled for analysis (Table 4). Although downward trends in estradiol levels and upward trends in fibrinogen and hs-CRP were observed, there was no statistically significance difference between levels of the biochemical markers before versus after the introduction of progesterone. An increase in weight after the introduction of progesterone was observed. After 6 months of progesterone therapy, 5 out of 10 subjects with an intact uterus achieved menarche. Pelvic ultrasounds obtained on 3 out of the 5 subjects who did not have menarche at the end of the study period demonstrated a prepubertal-sized uterus, and an endometrial stripe was only detected in 1/3 of those girls.

**Table 4:** Selected parameters (Mean ± SE) before and 6 months after introduction of progesterone (n = 8)

	**Before progesterone**	**On progesterone**
Height (cm)	152 ± 2.2	153.3 ± 2.2^*a*^
Weight (kg)	50.3 ± 7.7	53.1 ± 7.2^*a*^
Estradiol (pg/mL)	26 ± 10.4	11 ± 3.7
Estrone Sulfate (ng/dL)	204 ± 75	217 ± 115
HS-CRP (mg/L)	1.82 ± 1.42	3.83 ± 1.94
Fibrinogen Activity (mg/dL)	267 ± 32.2	310 ± 30.5
Antithrombin Activity (%)	109 ± 3.1	117 ± 7.2
Glucose (mg/dL)	81 ± 2.2	80 ± 1.6
ALT (U/L)	21 ± 5.5	26 ± 7.8
Triglycerides (mg/dL)	122 ± 23.6	111 ± 20
LDL (mg/dL)	84 ± 6.8	84 ± 5.7
IGF-1 (ng/mL)	416 ± 55	314 ± 56
Menses		50 %

^*a*^*p* < 0.05, difference in mean values between groups.

## Discussion

Estrogen replacement therapy is essential for girls with ovarian failure to induce puberty and deliver other estrogen-dependent effects. Currently, there is no consensus on the optimal route of estrogen delivery or dose. This 24 month prospective, randomized trial demonstrates that treatment with either transdermal or oral 17β estradiol resulted in higher estradiol levels and more effective feminization compared to oral conjugated estrogens at the doses selected but did not result in an otherwise different biochemical profile.

Our results are consistent with other studies of hypogonadal girls which have shown similar biochemical profiles between oral and transdermal estrogen treatment, but those studies were conducted over a shorter duration and limited to girls with TS. In a study comparing OCEE and TBE in increasing doses over 6 weeks in girls with TS treated with growth hormone, investigators concluded that there was no difference in plasma lipids, insulin, or fibrinogen levels with treatment using either form of estrogen [20]. In a one year study, girls over the age of 10 years with TS randomized to OCEE or TBE had no observed differences in serum levels of HDL, LDL or triglycerides [21]. These results are specifically important in girls with TS, who have increased incidence of hypertension (40% of population), an atherogenic lipid profile, and a higher risk of cardiovascular disease as adults (3 fold increase in mortality) [22-24].

In our study, serum estradiol concentrations rose over 18 months of estrogen therapy with all three forms of estrogen, consistent with the physiologic rise in estradiol through puberty. However, subjects treated with transdermal estrogen had significantly higher estradiol levels at the maximum estrogen dose. This result matches the more effective feminization demonstrated in girls treated with TBE compared to OCEE at similar dose equivalencies [21]. Our study also shows trends toward higher estrone and estrone sulfate levels during treatment with oral versus transdermal estrogen, but reaching statistical significance only at 12 months. The inconsistent statistical significance is likely due to the effect of small sample size as well as large variability in serum concentrations of estrone sulfate. This phenomenon is likely representative of the first-pass metabolism of oral estrogen through the liver and conversion of estradiol to estrone. These results are similar to other studies comparing the use of OBE and TBE in girls with TS [1,25]. Systemic absorption of estradiol via the transdermal route avoids initial metabolism through the liver, thus avoiding potential deleterious effects associated with OCEE. However, our study did not conclusively show differential effects on inflammatory markers or lipid or liver profiles. These differences in biochemical markers seen between the use of oral and transdermal estrogen in older women have not to date translated to adolescent girls.

We observed more effective feminization in girls treated with TBE and OBE compared to OCEE. This could be explained by more consistent levels of estrogen, mimicking spontaneous puberty [26]. The lack of menarche in over half of the subjects with intact uterus was likely due to insufficient estrogen exposure prior to the introduction of progesterone as demonstrated by the pre-pubertal size of the uterus on ultrasound in some of the girls. While rates of feminization were similar between girls treated with TBE and OBE, more girls treated with TBE achieved menarche. It is difficult to know if insufficient estrogenization of the uterus was due to the mode of estrogen delivery, the dose of estrogen, or due to patient adherence to the therapy. Torres-Santiago et al. demonstrated that doses in excess of our maximal estrogen doses (TBE 0.75-1 mg and OBE 2 mg) are required to maintain estradiol levels comparable to normally menstruating adolescent females, suggesting that dosage may have played a role in our results [1].

There are several limitations of our study including the small number of subjects, high variability of biochemical markers among patients, and the effects of underlying medical condition on inflammatory markers and liver and lipid metabolism. Due to our small sample size we were unable to separate subjects with TS or treated with growth hormone in our analysis. There was a trend towards higher IGF-1 levels with increasing doses of estrogen; however without separation of the subjects on rGH in the analysis the isolated effects of estrogen on IGF-1 would be difficult to discern. The large variability in estradiol and estrone sulfate levels could reflect the relationship between sample collection and administration of the medication, especially for estradiol levels in subjects on oral preparations (OCEE and OBE). Also, one cannot discount the potential effects of adherence to therapy when comparing the biochemical profiles of oral versus transdermal estrogen after 18 months of hormone therapy. The lack of differential effects of progesterone therapy is contrary to prior studies [16,17], but may be due to our small sample size, the potential variable of the timing of the sample collection in relation to the menstrual cycle, or the use of micronized progesterone rather than the more typical medroxyprogesterone.

## Conclusions

In summary, we observed higher estradiol levels and more effective feminization in girls treated with transdermal estrogen but no differences in other biochemical markers. Given widespread concerns about the use of oral conjugated estrogens, but no convincing evidence of differential biochemical profiles between oral and transdermal estradiol to date in adolescents, either oral or transdermal 17β estradiol could provide safe and effective alternatives to OCEE to induce puberty in girls. Larger prospective randomized trials that can account for the various underlying etiologies of ovarian failure are still required to determine the ideal regimen of estrogen delivery to adolescent females, but sufficient concerns surrounding the use of OCEE suggest the preferential use of oral or transdermal 17β estradiol.

## Abbreviations

TS: Turner Syndrome; HDL: High-density lipoprotein; LDL: Low-density lipoprotein; OCEE: Oral conjugated equine estrogen; TBE: Transdermal 17β estradiol; OBE: Oral 17β estradiol; E2: Estradiol; hs-CRP: High-sensitivity c-reactive protein; IGF-1: Insulin-like growth factor-1; BMT: Bone marrow transplant; AI: Androgen insensitivity syndrome.

## Competing interests

The authors declare that they have no competing interests.

## Authors’ contributions

SS participated in the study coordination, implementation, performed the statistical analysis, and wrote the first draft of the manuscript. NF conceived of the study and participated in its design and coordination and helped to draft and revise the manuscript. ED participated in study coordination and revising the manuscript. EN conceived of the study and participated in its design and coordination and helped to draft and revise the manuscript. All authors read and approved the final manuscript.
